# Immune reconstitution of human cytomegalovirus-specific T lymphocytes after allogeneic hematopoietic stem cell transplantation and their predictive role in reactivation

**DOI:** 10.3389/fimmu.2025.1464096

**Published:** 2025-03-12

**Authors:** Yuanqi Zhao, Sisi Zhen, Jiali Wang, Jieru Wang, Runzhi Ma, Li Liu, Aiming Pang, Rongli Zhang, Xin Chen, Weihua Zhai, Donglin Yang, Yi He, Mingzhe Han, Erlie Jiang, Sizhou Feng

**Affiliations:** ^1^ Fujian Institute of Hematology, Union Hospital, Fujian Medical University, Fuzhou, China; ^2^ State Key Laboratory of Experimental Hematology, National Clinical Research Center for Blood Diseases, Haihe Laboratory of Cell Ecosystem, Institute of Hematology & Blood Diseases Hospital, Chinese Academy of Medical Sciences & Peking Union Medical College, Tianjin, China; ^3^ Tianjin Institutes of Health Science, Tianjin, China

**Keywords:** cytomegalovirus, hematopoietic stem cell transplantation, immune monitoring, pre-emptive therapy, infection

## Abstract

Human cytomegalovirus (HCMV) is the most common virus to affect the recipients of allogeneic hematopoietic stem cell transplantation (allo-HSCT). HCMV reactivation increases the risk of secondary fungal and bacterial infections, as well as that of non-relapse mortality after allo-HSCT. This study investigates the post-transplantation reconstitution of HCMV-specific T cells and their role in the regulation of HCMV infections. Peripheral blood samples from CMV-seropositive allo-HSCT recipients (R+) and CMV-seropositive donors (D+) were collected from October 2019 to June 2021. Continuous quantification and function monitoring of CMV-specific CD4+/CD8+T lymphocytes were performed by flow cytometry after stimulation in an HCMV-pp65 pool *in vitro* and intracellular cytokine staining was performed. Plasma CMV–DNA was quantitatively detected by qPCR. The median age of patients (*n* = 131) was 34 (23–45) years. Post-transplantation HCMV reactivation occurred in 88 (67.2%) patients. HCMV-responsive CD4+T cells in non-HCMV reactivation patients was significantly higher than that in HCMV reactivation patients 30 days after transplantation (0.21 cells/μL vs 0.10 cells/μL; *P* = 0.005). Kaplan–Meier analysis showed that the incidence of HCMV reactivation in patients with low levels of HCMV-responsive CD4+T cells (<0.14 cells/μL) was significantly higher than that in patients with high levels of HCMV-responsive CD4+T cells (>0.14 cells/μL) (83.9% vs 54.7%; *P* < 0.001). Patients lacking HCMV-responsive CD8+T cells (<2 cells/μL) 60 days after allo-HSCT had a significantly higher risk of HCMV reactivation 100 days after transplantation (*HR* 9.932; *P* = 0.005). Patient age and the mononuclear cell-infusion level were correlated with the reconstructive levels of HCMV-responsive CD8+T cells 60 days after transplantation. Poor recovery of HCMV-responsive CD4+T cells 30 days post-transplantation is closely related to the risk of HCMV reactivation. The level of HCMV-responsive CD8+T cells 60 days post-transplantation is a good predictor for late-onset HCMV reactivation, which is particularly important for outpatient monitoring and management of patients with allo-HSCT, and facilitates individualized risk stratification for HCMV reactivation.

## 1 Introduction

Human cytomegalovirus (HCMV), also known as human Herpesvirus type 5, belongs to the herpesvirus beta subfamily (Betaherpesvirinae) and is highly specific to host cells. The host immune response can inhibit CMV replication, thereby preventing the onset and occurrence of various CMV diseases. However, it cannot clear the virus or stop its transmission. Primary CMV infection is usually asymptomatic in people with normal immune function. The virus remains in a lifelong latent state in such people (1). However, in immunocompromised patients, such as those who underwent allogeneic hematopoietic stem cell transplantation (allo-HSCT), have a primary infection, or experience a reactivation of CMV, it can lead to life-threatening complications.

Myeloid cells and their progenitor cells are important latent sites for CMV (2). The seropositivity of CMV has been recorded in approximately 83% of the global population. A seropositive rate of CMV of 50–99% has been recorded globally, although it varies depending on the geographical location and socioeconomic conditions. More than 95% of the seropositive rate of CMV has been noted among Chinese adults (1).

The infection rate of CMV after transplantation in high-risk patients, such as allo-HSCT recipients who are serologically positive for CMV and have not been provided with a preventive treatment, can reach up to 80% (3). CMV infection also increases the risk of secondary bacterial and fungal infections (4), as well as the incidence of graft-versus-host disease (GVHD) (5), thereby increasing non-relapsed mortality (NRM) (6).

The clinical manifestations of CMV infections are varied and atypical, including fever, thrombocytopenia, lymphadenectasis, or associated tissue invasive manifestations. CMV reactivation in immunocompromised patients can lead to CMV end-organ diseases (7), including pneumonia, hepatitis, encephalitis, retinitis, cystitis, and gastroenteritis. The lung and gastrointestinal tract are the most commonly involved organs in such conditions. The mortality rate of patients with CMV pneumonia is as high as 60% (3, 8).

CMV reactivation usually occurs within the first 100 days of allo-HSCT, although it has been recorded even after more than 100 days in some patients. The prediction of late-onset CMV reactivation is particularly important for outpatient monitoring and management of patients with allo-HSCT. Notably, conventional prophylactic anti-CMV therapy is not performed in patients undergoing HSCT because of drug-related myelosuppression side effects. At present, the monitoring of CMV after allo-HSCT is mainly performed using PCR to detect the replication of CMV–DNA in peripheral blood (9). However, as the decision to begin pre-emptive treatment solely depends on the CMV–DNA assay, it may lead to delayed treatment in some patients, which may in turn give rise to the CMV disease. It may also lead to over-treatment in some patients, resulting in side effects owing to the use of excessive drugs.

CMV can reactivate from its latent state under the influence of changes in the host immune status. Intermittent subclinical viral reactivation is controlled by the host immune system and can drive CMV-specific changes in T cells in the host peripheral blood (10, 11). The host CMV-specific T lymphocyte immune response plays a critical role in controlling viral infections and cancer prevention (10–12). The majority of post-allo-HSCT CMV infections typically occur within 100 days of neutrophil engraftment and transplantation. More than 70% of the recipients of allo-HSCT who exhibit seropositivity for CMV are at a high risk of developing CMV reactivation within the first 100 days of transplantation (13). Therefore, monitoring the early CMV immune function after allo-HSCT is particularly important.

This study aims to investigate the influence of donor CMV-specific T lymphocytes on CMV immune reconstitution after allo-HSCT and the regulatory role of CMV-specific T cells in response to CMV reactivation after transplantation. This study uses the CMV-pp65 peptide pool to stimulate lymphocytes *in vitro* and quantitatively analyzes CMV-specific CD4+/CD8+T cells along with a multifunctional parameter for intracellular cytokine detection to dynamically monitor the CMV-specific CD4+T and CD8+T lymphocyte immune responses of the donors and receivers of allo-HSCT. The study results may provide an effective scientific basis for elucidating CMV reactivation after allo-HSCT and formulating risk strategies for predicting late CMV and refractory CMV reactivations.

## 2 Materials and methods

### 2.1 Research object

Peripheral blood samples from the recipients of CMV-seropositive allo-HSCT (R+) and CMV-seropositive donors (D+) were collected from October 2019 to June 2021. Continuous quantification and functional monitoring of CMV-specific T lymphocytes were performed through colorful flow cytometry after *in vitro* stimulation and cell culture. CMV–DNA testing was carried out at least once a week for the first 3 months after transplantation. The following criteria were employed to exclude the patients: (1) insufficient follow-up time (<150 days); and (2) occurrence of CMV reactivation within 28 days of transplantation.

The patients were grouped based on the clinical presentation and auxiliary examination results (7, 14, 15). CMV reactivation was defined as the detection of plasma CMV–DNA ≥1000 copies/mL by qPCR. Recurrent CMV reactivation was defined as the occurrence of CMV reactivation at least 4 weeks after CMV–DNA turned negative following antiviral therapy. Late CMV reactivation was defined as CMV reactivation occurring 100 days after allo-HSCT. CMV end-organ diseases were defined according to the criteria published by the CMV Drug Development Forum in 2018. Refractory CMV reactivation (RCMV) was defined as CMV–DNA elevation or progression to CMV disease after 2 weeks of regular antiviral therapy.

None of the patients received prophylactic anti-CMV therapy. Pre-emptive anti-CMV therapy with ganciclovir or foscarnet sodium was started when CMV reactivation occurred and was continued until CMV–DNA came out negative for two consecutive times. This study was approved by the Ethics Committee of the Institute of Hematology and Blood Diseases Hospital, Chinese Academy of Medical Sciences.

### 2.2 HCMV-pp65 peptides

Based on the known HCMV (AD169 strain)-restricted epitopes, we designed 15 peptide sequences, comprising 11 amino acid overlaps, spanning the full length of the HCMV-PP65 antigen. These sequences can induce the reactivation and proliferation of CMV-specific T cells. This peptide pool consists of 138 peptides (15-mers with 11 amino acid overlaps) synthesized and purified by JPT Peptide Technologies (Germany). The purity of all peptides was >90%, as determined through high-performance liquid chromatography (HPLC). To prepare an overlapping peptide storage solution before use, a bottle of sealed freeze-dried peptide powder was opened after bringing it down to room temperature. Then, 50 μL of DMSO was added until it completely dissolved. Finally, the resulting solution was immediately divided into smaller portions and stored below -20°C. The storage solution was diluted with RPMI 1640 (1:50) to the final concentration.

### 2.3 Collection and preparation of peripheral blood mononuclear cells

PBMCs were obtained from heparinized whole blood through lymphocyte isolation via density gradient centrifugation and cryopreserved for further analysis. Cell viability was evaluated through Trypan blue exclusion.

### 2.4 T cell stimulation *in vitro*

PBMCs were resuspended with RPMI 1640 containing 10% FBS (Gibco) at 1.0–2.5×10^6^/mL of concentration. The cell suspension was incubated in 24-well plates in the presence of 1 μg/mL of costimulatory mAb to CD28 and CD49d (BD Pharmingen). For CMV-specific T cell stimulation, the HCMV-pp65 peptide pool with a final concentration of 1 μg peptide/mL was added to stimulate T cells. A final concentration of 1 ng/mL of PMA was added to the positive control group. Costimulatory antibodies alone were added to the negative control group. Both groups were maintained at 37°C for 1 h. Brefeldin A (eBioscience), at a final concentration of 10 μg/mL, was added to each group. An additional 5 h of incubation was conducted, which was then terminated by centrifugation at 1500 rpm at 4°C for 6 min. The cells were incubated with diluted Zombie Aqua™ Fixable solution (Biolegend) at room temperature, in the dark, for 15–30 min to assess live vs. dead status of cells. After extracellular staining, the cells were fixed and permeabilized using a fixation/permeabilization solution kit (BD Pharmingen). Intracellular cytokine staining was carried out and the simultaneous expression of surface markers and intracellular cytokine was assessed.

### 2.5 Cytokine flow cytometry assays

Before conducting flow cytometry, extracellular staining and intracellular cytokine staining were performed using the following fluorescent labeled anti-human antibodies: CD3 (APC-Cy7), CD4 (BV421), CD8 (PerCP) (Biolegend) and IFN-γ (FITC), TNF-α (PE-Cy7), CCl_4_ (PE), and IL-2 (APC) (eBioscience). As part of the control-level setting and gate setting strategy, multicolor flow cytometry was performed on a canto II flow cytometer. FlowJo software was used to analyze the data obtained through flow cytometry. Next, the lymphocyte groups were gated in FSC/SSC, CD3/CD8, and CD3/CD4 double positive cell groups. Based on the results obtained, cell subgroups with a positive expression of cytokines were gated. CMV-specific T cell function was detected using the method described above. The actual frequency of CMV-specific T cells was calculated based on the difference between the CMV-pp65 group and the negative control group. CMV-specific CD4+T and CD8+T cell absolute counts were determined using the total lymphocyte count, the percentage of CD4+ and CD8+T cells in the lymphocyte and CD3+ gates, and the percentage of cytokine-producing cells. CMV-specific T cells that recognize CMV-pp65 overlapping peptides and express any of the cytokines in IFN-γ, IL-2, TNF-α, and CCl_4_ represent CMV-responsive T cells.

### 2.6 Statistical analyses

Continuous variables were expressed in median and interquartile intervals (IQRs), while categorical variables were expressed in counts and percentages. Chi-square test was used for the univariate analysis of categorical variables. Mann–Whitney U test was used for continuous variable comparison. The Kaplan–Meier curve was used to estimate the cumulative probability of CMV reactivation. The log-rank test was used to compare differences between the groups. Cox proportional hazard regression models were used to estimate the hazard ratio (HR) of the intergroup CMV reactivation outcome. Variables with *P* values <0.05 determined through the univariate analysis were subsequently included in the multivariate logistic regression model. *P* < 0.05 on both sides was considered statistically significant. Data image generation and statistical analysis were performed using GraphPad Prism 7.0 and SPSS 23.0 software.

## 3 Results

### 3.1 Clinical outcomes

In this study, 131 allo-HSCT recipients (men = 44.3%) were included, with a median age of 34 (23–45) years. None of the patients received preventive anti-CMV therapy after allo-HSCT. The baseline characteristics of the patients are listed in **Table 1**. The median follow-up time was 232.5 (152.0–546.0) days. The median time for the first CMV reactivation was 40.0 days after HSCT (IQR, 32.3–50.0). At least one post-transplant CMV reactivation was noted in 88 (67.2%) patients, the median peak value of CMV–DNA was 5151.0 copies/mL (IQR, 2387.5–9234.0), and the median duration of CMV reactivation was 15 days (IQR, 7.5–19.0). The CMV–DNA loading peak of 56 patients with RCMV was significantly higher than that of patients without RCMV (7624.0 (IQR, 4816.5–15327.0) copies/mL vs 2050.5 (IQR, 1393.0–4390.0) copies/mL; *P* < 0.001).

**Table 1:** Baseline characteristics of patients.

Characteristic	*N* = 131
Median age (IQR)	34.0 (23.0-45.0)
Gender, male, *n* (%)	58.0 (44.3)
Disease type, *n* (%)	
AML	54.0 (41.2)
ALL	39.0 (29.8)
MDS	30.0 (22.9)
AA	8.0 (6.1)
Transplantation type, *n* (%)	
Unrelated donor	10.0 (7.6)
Matched related donor	35.0 (26.7)
Haploidentical donor	86.0 (65.6)
Prior ATG use, *n* (%)	107.0 (81.7)
Prior high-dose glucocorticoid use, *n* (%)	35.0 (26.7)
Grade III–IV aGVHD, *n* (%)	21.0 (16.0)
Leukemia relapse after allo-HSCT, *n* (%)	21.0 (16.0)
CMV reactivation after allo-HSCT, *n* (%)	88.0 (67.2)
Time from allo-HSCT to the first CMV reactivation, days, median (IQR)	40.0 (32.3-50.0)
CMV–DNA peak value, median (IQR), copies/mL	5151.0 (2387.5-9234.0)
Duration of the first CMV reactivation, median (IQR), days	15.0 (7.5-19.0)
Recurrent CMV reactivation, *n* (%)	43.0 (32.8)
Late-onset CMV reactivation, *n* (%)	24.0 (18.3)
Follow-up, median (IQR)	232.5 (152.0-546.0)
Days to neutrophil engraftment, median (IQR)	12.0 (11.0-14.0)
Days to platelet engraftment, median (IQR)	15.0 (13.0-18.8)
MNC dose, 10^8^/kg, median (IQR)	10.45 (8.55-13.74)
CD34+ cell dose, 10^6^/kg, median (IQR)	2.59 (2.30-3.08)
Donor and recipient of the same blood type, *n* (%)	79.0 (60.3)

AML, acute myeloid leukemia; ALL, acute lymphoblastic leukemia; MDS, myelodysplastic syndrome; AA, aplastic anemia; ATG, antithymocyte globulin; GVHD, graft-versus-host disease; MNC, mononuclear cell; high-dose glucocorticoid (prednisone > 1 mg/kg/d), IQR, interquartile range.

### 3.2 Functional reconstitution of CMV-specific T cells in the early stage after allo-HSCT

**Figure 1:**
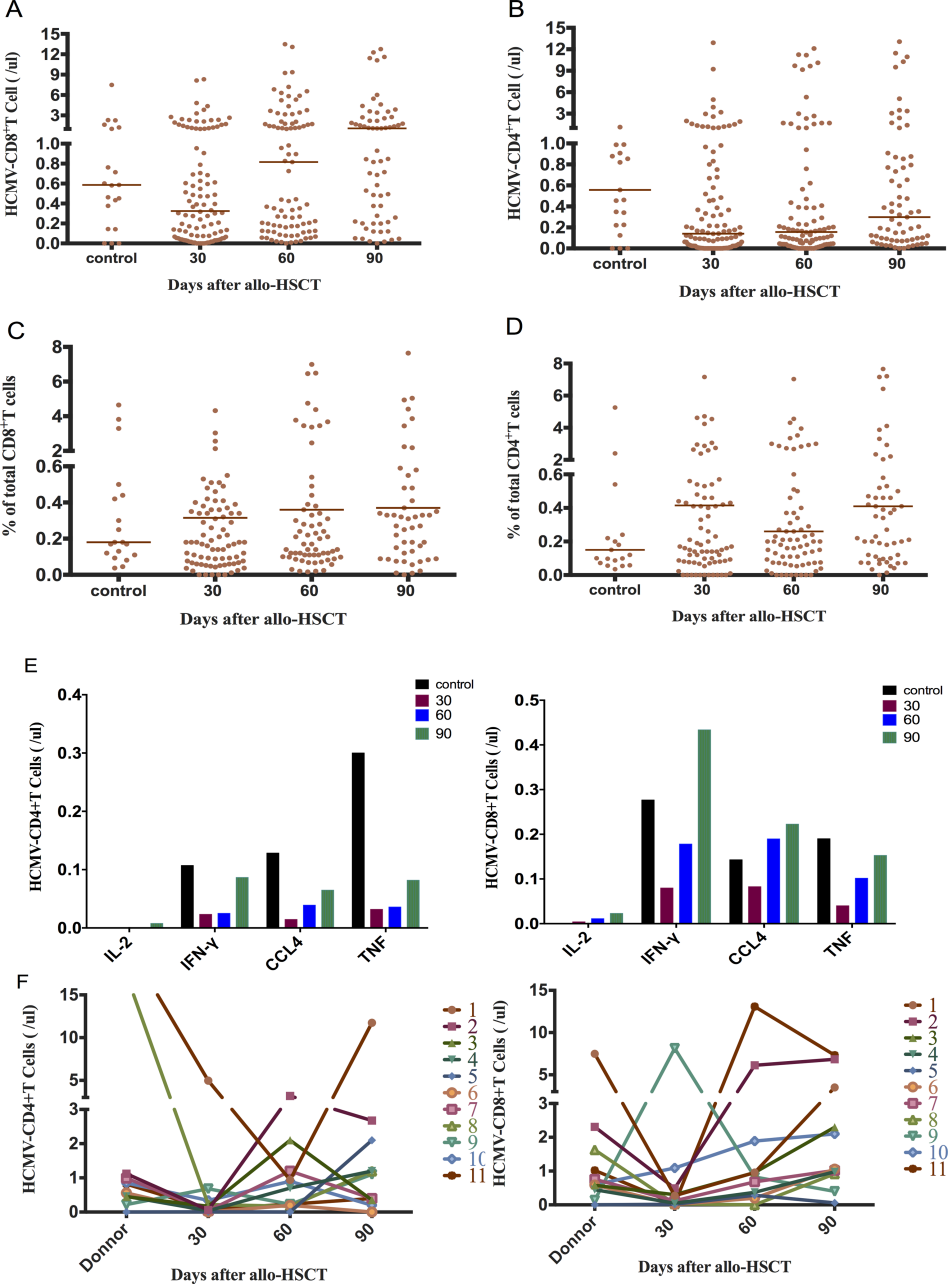
Early reconstitution of CMV-responsive CD4+T and CD8+T lymphocytes after allo-HSCT. **(A)** CMV-responsive CD8+T recovered to the normal group level at 30+ days (0.32 cells/μL vs 0.59 cells/μL; *P* = 0.282). It reached 0.82 cells/μL at 60+ days, which was significantly higher than that at 30+ days (*P* = 0.007). **(B)** CMV-responsive CD4+T cells recovered slowly, returning to normal control levels at 90+ days (0.30 cells/μL vs 0.56 cells/μL; *P* = 0.139). **(C)** Proportion of CMV-responsive CD8+T cells in total CD8+T cells. **(D)** Proportion of CMV-responsive CD4+T cells in total CD4+T cells. **(E)** Changes in the distribution of CMV-responsive CD4+ and CD8+T cell in single-cytokine responses. The cytokines secreted by CMV-responsive CD8+T cells were mainly IFN-γ in the normal control group 90 days after transplantation. CCl_4_ was mainly secreted 30 days after transplantation. **(F)** Relationship between donor and recipient CMV-responsive CD4+ and CD8+T cell reconstitution 30, 60, and 90 days after allo-HSCT. The horizontal lines in the scatter plot represent the median level of each group.

We analyzed the immune response of peripheral blood CD4+T and CD8+T cells to the CMV-PP65 peptide pool in 131 recipients of CMV-seropositive allo-HSCT and 22 normal control subjects. CMV-specific T cells that recognize CMV-pp65 and secrete cytokines, including IFN-γ, IL-2, TNF-α, and CCL4, are defined as CMV-responsive T cells. The CMV-responsive CD8+T cell count 30 days after transplantation was not significantly different from that of the normal control group (0.32 (IQR, 0.06–1.22) cells/μL vs 0.59 (IQR, 0.26–1.43) cells/μL; *P* = 0.282). A significant increase was observed in the count of CMV-responsive CD8+T lymphocytes 60 days after transplantation compared to that observed 30 days after transplantation (0.82 (IQR, 0.17–3.25) cells/μL vs 0.32 (IQR, 0.06–1.22) cells/μL; *P* = 0.007) (**Figure 1A**). There was no significant change in the proportion of CMV-responsive CD8+T cells 90 days after allo-HSCT (**Figure 1C**).

CMV-responsive CD4+T cells gradually recovered their numbers after allo-HSCT, although it was significantly lower than that of the normal control group 30 days after transplantation (0.14 cells/μL vs 0.56 cells/μL; *P* = 0.016). The CMV-responsive CD4+T cell count reached the control level 90 days after transplantation (*P* = 0.139) (**Figure 1B**). The proportion of CMV-responsive CD4+T cells did not change significantly within 90 days of allo-HSCT and was not significantly different from that of the normal control (**Figure 1D**). The CMV-responsive CD8+T cells of the normal control group mainly secreted the IFN-γ cytokine after 90 days of transplantation, while CCl_4_ was mainly secreted 30 days after transplantation (**Figure 1E**).

We performed CMV-specific immunoassay on 11 pairs of donors and recipients (**Figure 1F**). No significant correlation was observed between the levels of CMV-responsive CD4+T and CD8+T cells in recipients at 30, 60, and 90 days after allo-HSCT and those of the corresponding donors (Spearman correlation test).

### 3.3 Cellular immunity and control of CMV reactivation

#### 3.3.1 Memory CD8+T cells are associated with the control of CMV reactivation

**Figure 2:**
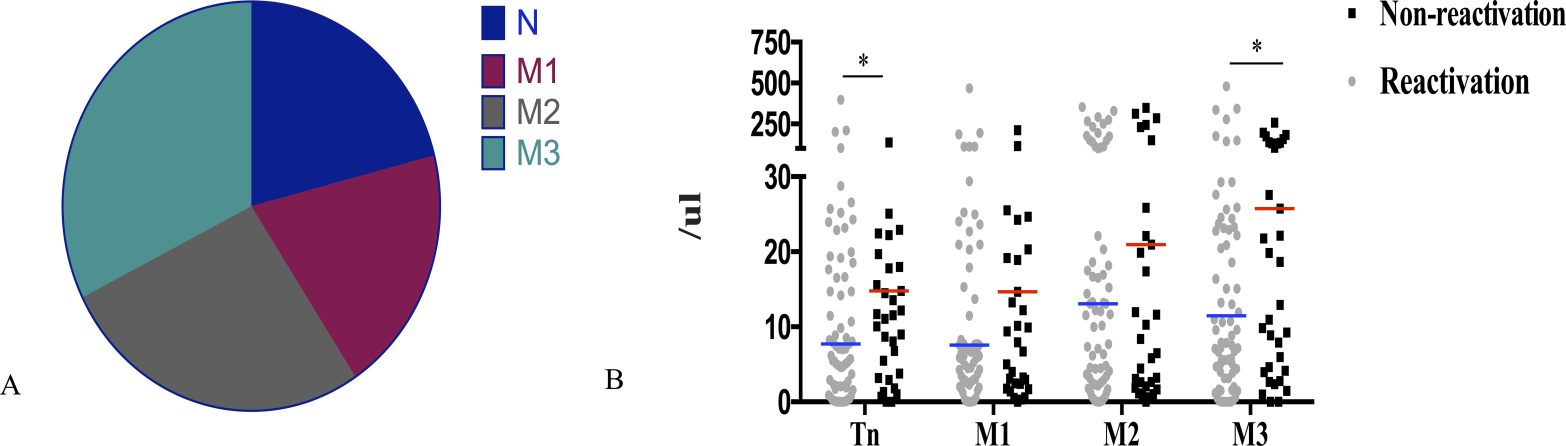
Association of CD8+T cell subsets 30 days post-allo-HSCT with CMV reactivation. **(A)** Percentage distribution of CD8+T cell subsets 30 days after allo-HSCT. **(B)** Patients without CMV reactivation after allo-HSCT had significantly higher naive T cells (Tn) and terminally differentiated effector memory T cells (M3) 30 days after transplantation than that in patients without CMV reactivation (14.78 cells/μL vs 7.28 cells/μL; *P* = 0.022; 25.76 cells/μL vs 10.86 cells/μL; *P* = 0.017); *P < 0.05. The horizontal lines in the scatter plot correspond to the median level of each group of IQR; Tn, CD27+CD45RA+; M1, CD27+CD45RA-; M2, CD27-CD45RA-; M3, CD27-CD45RA+.

After 30 days of allo-HSCT, the CD8+T cells mainly comprised terminally differentiated effector memory T cells (TEMRA) (32.5%, **Figure 2A**). Patients with CMV reactivation had significantly lower naive T cells (Tn) and terminally TEMRA 30 days after allo-HSCT than those without CMV reactivation (*P* = 0.022, *P* = 0.017; **Figure 2B**).

#### 3.3.2 Level of CMV-specific CD4+T cell immune response 30 days post-transplantation predicted the risk of CMV reactivation

**Figure 3:**
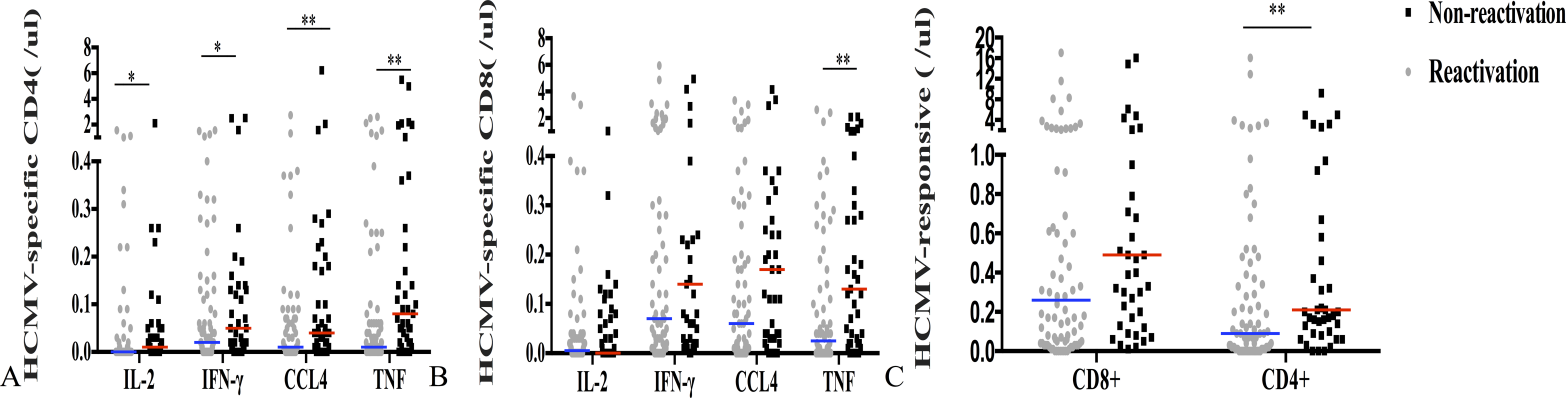
CMV-specific T cell immune response and CMV reactivation 30 days after allo-HSCT. **(A)** CMV-specific CD4+T cell levels that produce IFN-γ, IL-2, TNF-α, or CCl_4_ in both reactivated and non-reactivated groups. **(B)** CMV-specific CD8+T cells that produce IFN-γ, IL-2, TNF-α, or CCl_4_ were identified in both reactivated and non-reactivated groups. **(C)** CMV-responsive CD4+ and CD8+T cells. The horizontal line represents the median level of each group. **P < 0.01, *P < 0.05.

**Figure 4:**
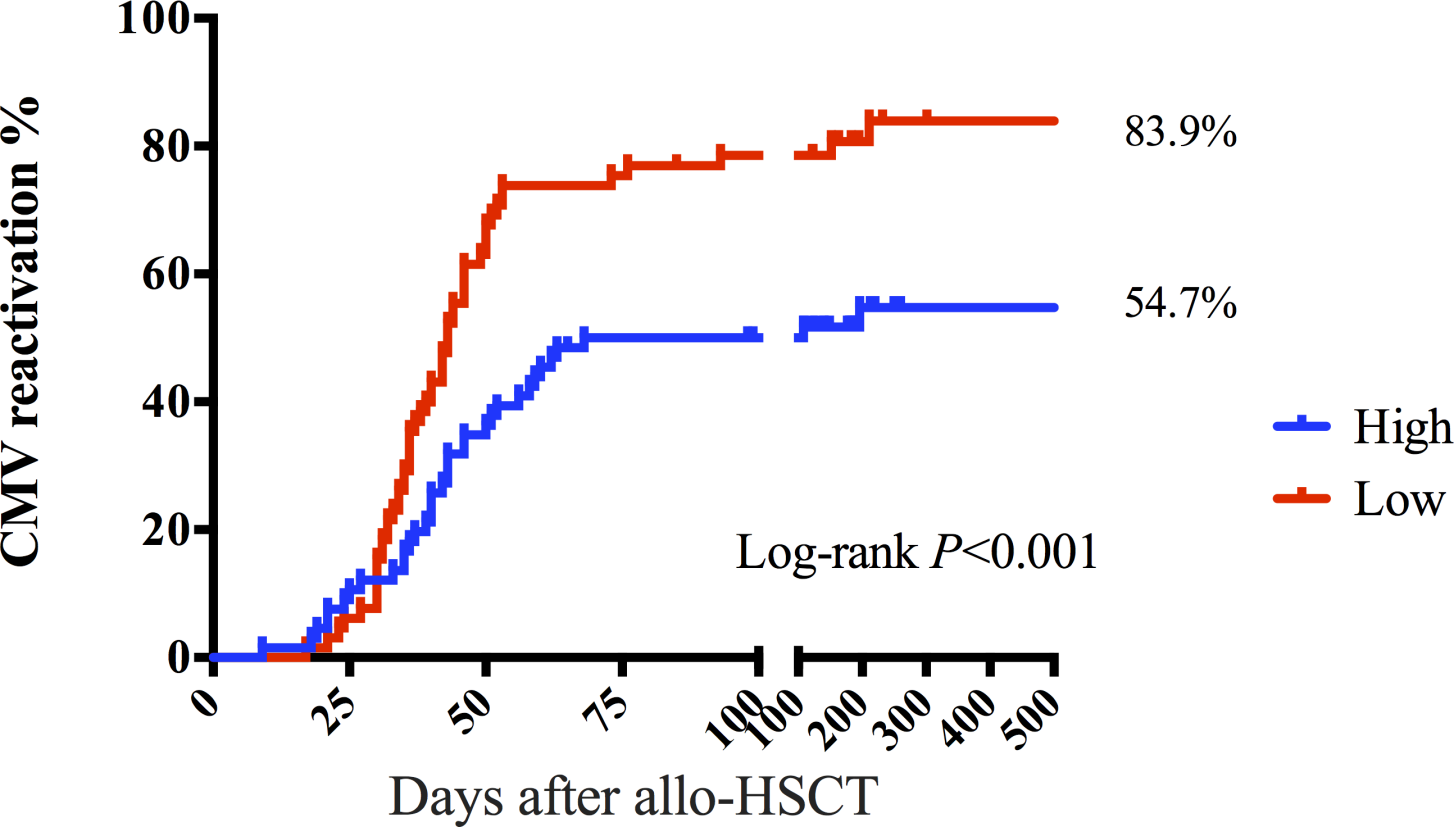
CMV-responsive CD4+T lymphocytes distinguish the risk of CMV reactivation after allo-HSCT. The cumulative incidence of CMV reactivation was 54.7% in the group with >0.14 CD4+T cells/μL 30 days after allo-HSCT. The cumulative incidence of CMV reactivation was 83.9% in the group of CMV-responsive CD4+T cells (<0.14 cells/μL) 30 days after allo-HSCT. The *P*-value corresponds to the log-rank test results.

There was no significant difference in the count of CMV-responsive CD8+T cells that produced IFN-γ, IL-2, or CCl_4_ between patients with and without CMV reactivation (*P* = 0.235, *P* = 403, and *P* = 0.144). However, the number of CD4+T or CD8+T cells producing TNF-α was significantly different between the two groups (*P* = 0.005, *P* = 0.005) (**Figures 3A, B**). The median CMV-responsive CD4+T cell level in patients without CMV reactivation was significantly higher than that in patients with CMV reactivation (0.21 (IQR, 0.10–1.03) cells/μL vs 0.10 (IQR, 0.01–0.48) cells/μL; *P* = 0.005) (**Figure 3C**). We further grouped patients based on their +30-day CMV-responsive CD4+T cell levels. The results showed that the transplant type (unrelated, haploidentical), ATG, and III–IV aGVHD are significantly associated with delayed reconstitution of CMV-responsive CD4+T cells 30 days after transplantation (**Table 2**). Patients with CMV-responsive CD4+T cells (<0.14 cells/μL) 30 days after transplantation have a higher risk of CMV reactivation (hazard ratio, [HR] 2.139, 95%CI: 1.390–3.293; *P* < 0.001). Kaplan–Meier analysis showed that the cumulative incidence of CMV reactivation in patients with low levels of CMV-responsive CD4+T cells (<0.14/μL) is significantly higher than that in those with high levels of such cells (>0.14/μL) (*P* < 0.001) (**Figure 4**). In patients with high levels of CMV-responsive CD4+T cells 30 days after allo-HSCT, CMV reactivation is associated with underlying diseases, transplant types, immunosuppressive uses, and CD34+ infusion levels (**Table 3**).

**Table 2:** Factors influencing CMV-responsive CD4+T cell reconstitution 30 days after allo-HSCT.

Characteristic	High levels of CMV–CD4 (*n* = 66)	Low levels of CMV–CD4 (*n* = 65)	*P*-value
Median age, (IQR)	35.0 (23.8-46.3)	34.0 (22.5-44.0)	0.587
Gender, male	28.0 (42.4)	30.0 (46.2)	0.726
Disease type, *n* (%)			0.560
AML	29.0 (43.9)	25.0 (38.5)	
ALL	20.0 (30.3)	19.0 (29.2)	
MDS	12.0 (18.2)	18.0 (27.7)	
AA	5.0 (7.6)	3.0 (4.6)	
Transplantation type, *n* (%)			0.003
Unrelated donor	3.0 (4.5)	7.0 (10.8)	
Matched related donor	26.0 (39.4)	9.0 (13.8)	
Haploidentical donor	37.0 (56.1)	49.0 (75.4)	
Prior ATG use, *n* (%)	48.0 (72.7)	59.0 (90.8)	0.012
Prior high-dose glucocorticoid, *n* (%)	10.0 (15.2)	25.0 (38.5)	0.003
Grade III–IV aGVHD, *n* (%)	4.0 (6.1)	17.0 (26.2)	0.002
Donor and recipient of the same blood type, *n* (%)	41.0 (63.1)	38.0 (59.4)	0.720
MNC dose, 10^8^/kg, median (IQR)	10.47 (8.62-15.23)	10.45 (8.31-13.00)	0.512
CD34+ cell dose, 10^6^/kg, median (IQR)	2.50 (2.29−3.01)	2.68 (2.34-3.12)	0.155

High levels of CMV–CD4 refer to CMV-responsive CD4+T cells (>0.14 cells/μL).

AML, acute myeloid leukemia; ALL, acute lymphoblastic leukemia; MDS, myelodysplastic syndrome; AA, aplastic anemia; ATG, antithymocyte globulin; GVHD, graft-versus-host disease; MNC, mononuclear cell; high-dose glucocorticoid (prednisone > 1 mg/kg/d), IQR, interquartile range.

**Table 3:** Factors influencing CMV reactivation in patients with high levels of CMV-responsive CD4+T cells 30 days after allo-HSCT.

Characteristic	CMV-reactivation group	Non-CMV-reactivation group	*P*-value
	(*n* = 35)	(*n* = 31)	
Median age, (IQR)	37.0 (24.0)	29.0 (22.0-41.0)	0.055
Gender, male, *n* (%)	14.0 (40.0)	14.0 (45.2)	0.804
Disease type*, n* (%)			0.041
AML	18.0 (51.4)	11.0 (35.5)	
ALL	5.0 (14.3)	15.0 (48.4)	
MDS	9.0 (25.7)	3.0 (9.7)	
AA	3.0 (8.6)	2.0 (6.5)	
Transplantation type, *n* (%)			<0.001
Unrelated donor	1.0 (2.9)	2.0 (6.5)	
Matched related donor	6.0 (17.1)	20.0 (64.5)	
Haploidentical donor	28.0 (80.0)	9.0 (29.0)	
Prior ATG use, *n* (%)	32.0 (91.4)	16.0 (51.6)	0.001
Prior high-dose glucocorticoid, *n* (%)	9.0 (25.7)	1.0 (3.2)	0.015
III–IV aGVHD *n* (%)	3.0 (8.6)	1.0 (3.2)	0.616
Leukemia relapse after allo-HSCT	3.0 (8.6)	6.0 (19.4)	0.287
MNC dose, 10^8^/kg, median (IQR)	12.27 (8.69-15.51)	10.00 (8.44-11.69)	0.050
CD34+ cell dose, 10^6^/kg, median (IQR)	2.60 (2.42-3.10)	2.34 (2.17-2.59)	0.005
Donor and recipient of the same blood type	22.0 (62.9)	19.0 (63.3)	0.968

High levels of CMV–CD4 indicate the presence of CMV-responsive CD4+T cells (>0.14 cells/μL).

**Figure 5:**
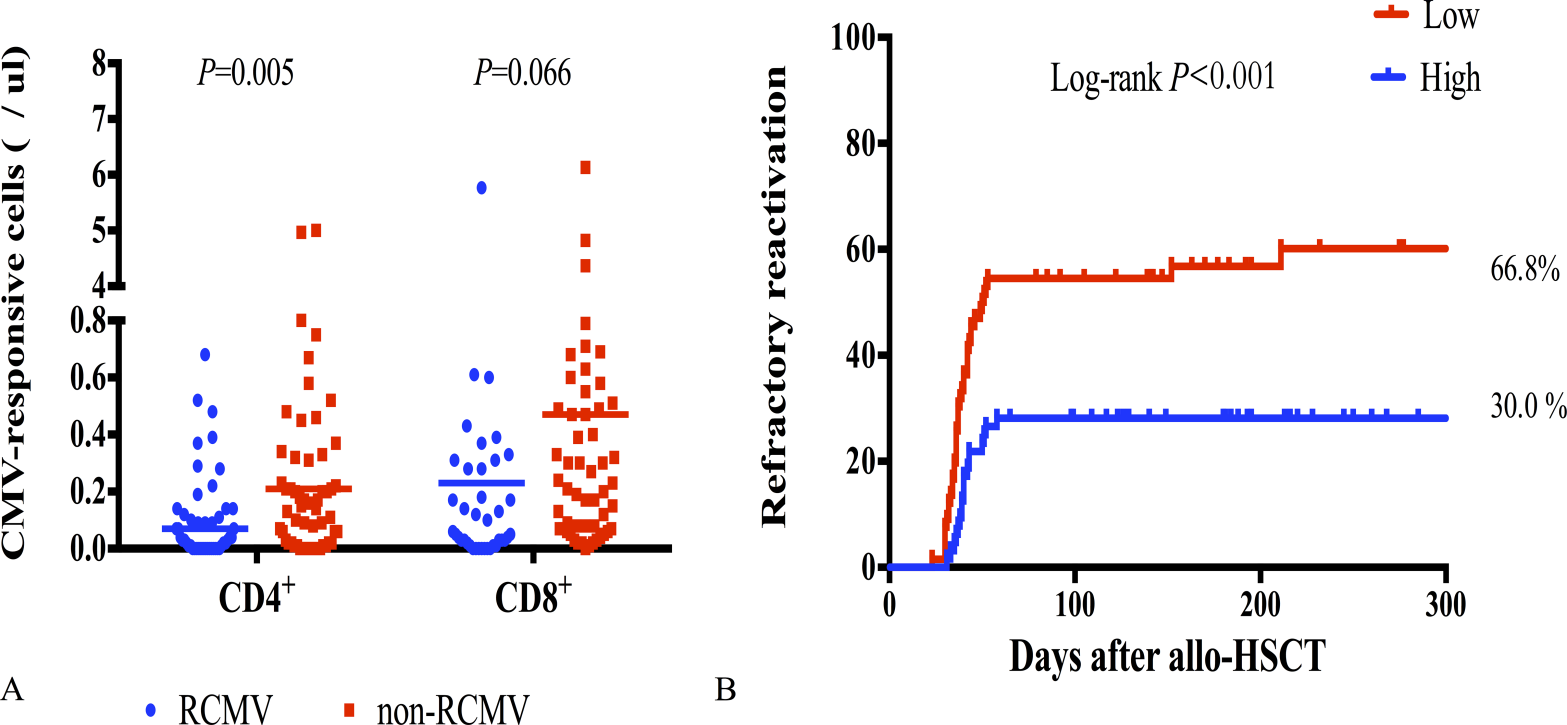
CMV-responsive T cells with refractory CMV reactivation. **(A)** CMV-responsive CD4+ and CD8+T cell counts 30 days after allo-HSCT in patients with RCMV (*n* = 56) and without RCMV (*n* = 75); RCMV, refractory CMV; horizontal line in the scatter plot indicates the median level. **(B)** Incidence of RCMV after allo-HSCT significantly increased in patients with low levels of CMV-responsive CD4+T cells (<0.14 cells/μL) 30 days after allo-HSCT, with a cumulative incidence of 66.8%.

Next, we compared CMV-specific cellular immunity levels in patients with refractory CMV reactivation (RCMV) after allo-HSCT with those without RCMV to predict the occurrence of RCMV after pre-emptive therapy. RCMV was noted in 56 patients, with a median time of occurrence of 38.5 days after allo-HSCT (IQR, 32.3–43.8). CMV-responsive CD4+T cell levels 30 days post-transplantation were correlated with the subsequent RCMV. CMV-responsive CD4+T cell levels were significantly lower in the RCMV group than they were in the non-RCMV group (0.21 cells/μL vs 0.07 cells/μL; *P* = 0.005) (**Figure 5A**). The cumulative incidence of post-transplantation RCMV was significantly lower in the group with a higher level of CMV-responsive CD4+T cells (>0.14 cells/μL) 30 days post-allo-HSCT than it was in the group with a lower level of CMV-responsive CD4+T cells (<0.14/μL) (66.8% vs 30.0%; *P* < 0.001) (**Figure 5B**).

#### 3.3.3 Level of CMV-responsive T cells 60 days after allo-HSCT can be used as a sensitive predictor of late-onset CMV reactivation

CMV reactivation usually occurs in the first 100 days after allo-HSCT, although some patients do experience CMV reactivation even after more than 100 days. Therefore, it is crucial to predict late CMV reactivation for outpatient monitoring and managing patients with allo-HSCT. Of the 92 patients ultimately included in the CMV-specific T cell analysis 60 days after transplantation, 21 had late-onset CMV reactivation. The CMV-responsive CD4+T cells were almost undetectable in patients with late-onset CMV reactivation 60 days after allo-HSCT. The median level of CMV-responsive CD8+T cells was 0.20/μL. The median levels of CMV-responsive CD4+ and CD8+T cells were 0.20 and 1.00/μL, respectively, in patients without late-onset CMV reactivation. Patients without late-onset CMV reactivation had significantly higher levels of CMV-responsive CD4+ and CD8+T cells 60 days after allo-HSCT than those in patients with late-onset CMV reactivation (*P* = 0.009, *P* = 0.003). Univariate analysis showed that age and mononuclear cell (MNC) infusion levels are associated with the reconstructive levels of CMV-responsive CD8+T cells 60 days after transplantation (**Table 4**).

**Table 4:** Factors influencing the reconstitution of CMV-responsive CD8+T cells 60 days after allo-HSCT.

Characteristic	High levels of CMV–CD8	Low levels of CMV–CD8	*P*-value
	(*n* = 27)	(*n* = 65)	
Median age, (IQR)	37.0 (34.0-47.0)	33.0 (19.0-44.5)	0.011
Gender, male	11.0 (40.7)	28.0 (43.1)	0.832
Disease type, *n* (%)			0.181
AML	10.0 (37.0)	25.0 (38.5)	
ALL	11.0 (40.7)	17.0 (26.2)	
MDS	5.0 (18.5)	19.0 (29.2)	
AA	1.0 (3.7)	4.0 (6.2)	
Transplantation type, *n* (%)			0.380
Unrelated donor	3.0 (11.1)	5.0 (7.7)	
Matched related donor	8.0 (29.6)	12.0 (18.5)	
Haploidentical donor	16.0 (59.3)	48.0 (73.8)	
Prior ATG use, *n* (%)	24.0 (88.9)	56.0 (86.2)	0.723
Prior high-dose glucocorticoid, *n* (%)	7.0 (25.9)	25.0 (38.5)	0.338
Grade III–IV aGVHD, *n* (%)	4.0 (14.8)	19.0 (29.2)	0.190
Donor and recipient of the same blood type, *n* (%)	21.0 (77.8)	38.0 (58.5)	0.095
MNC dose, 10^8^/kg, median (IQR)	12.21 (10.13-15.71)	10.00 (8.00-13.52)	0.008
CD34+ cell dose, 10^6^/kg	2.51 (2.23-3.34)	2.64 (2.35-3.28)	0.752
Days to neutrophil engraftment, median (IQR)	12.0 (11.0-14.0)	12.0 (11.0-14.0)	0.314
Days to platelet engraftment, median (IQR)	14.0 (13.0-17.5)	16.0 (14.0-20.0)	0.265
CMV reactivation occurred within 60 days after allo-HSCT, *n* (%)	17.0 (63.0)	48.0 (73.8)	0.322

High levels of CMV–CD8 indicate the presence of CMV-responsive CD8+T cells (>2.00 cells/μL) 60 days after allo-HSCT.

AML, acute myeloid leukemia; ALL, acute lymphoblastic leukemia; MDS, myelodysplastic syndrome; AA, aplastic anemia; ATG, antithymocyte globulin; GVHD, graft-versus-host disease; MNC, mononuclear cell.

Of the 27 patients with >2.00 CMV-responsive CD8+T cells/μL 60 days after allo-HSCT, only one developed late-onset CMV reactivation. This patient developed leukemia relapse 120 days after allo-HSCT. However, late-onset CMV reactivation occurred in up to 30.8% (20/65) of patients lacking CMV-responsive CD8+T cells. Among patients with low levels of CMV-responsive CD8+T cells 60 days after allo-HSCT, there were differences in the underlying disease, aGVHD levels, high-dose hormone usage, and underlying disease relapse between groups with and without late-onset CMV reactivation (**Table 5**).

**Table 5:** Univariate analysis of late-onset CMV reactivation in patients with low levels of CMV-responsive CD8+T cells 60 days after allo-HSCT.

Characteristic	Late-onset CMV reactivation	Non-late-onset CMV reactivation	*P*-value
	(*n* = 20)	(*n* = 45)	
Median age, (IQR)	33.5(15.8-49.8)	29.0(21.0-42.5)	0.680
Gender, male	6.0(30.0)	22.0(48.9)	0.184
Disease type, *n* (%)			0.019
AML	11.0(55.0)	14.0(31.1)	
ALL	1.0(5.0)	16.0(35.6)	
MDS	8.0(40.0)	11.0(24.4)	
AA	0	4.0(8.9)	
Transplantation type, *n* (%)			0.391
Unrelated donor	1.0(5.0)	4.0(8.9)	
Matched related donor	2.0(10.0)	10.0(22.2)	
Haploidentical donor	17.0(85.0)	31.0(68.9)	
Prior ATG use, *n* (%)	19.0(95.0)	37.0(82.2)	0.255
Prior high-dose glucocorticoid, *n* (%)	13.0(65.0)	12.0(26.7)	0.005
Grade III–IV aGVHD, *n* (%)	12.0(60.0)	7.0(15.6)	0.001
Relapse after allo-HSCT, *n* (%)	7.0(35.0)	3.0(6.7)	0.007
MNC dose, 10^8^/kg, median (IQR)	9.71(7.00-11.45)	10.00(8.19-14.78)	0.095
CD34+ cell dose, 10^6^/kg	2.69(2.40-2.78)	2.63(2.31-3.39)	0.994
Donor and recipient of the same blood type, *n* (%)	14.0(70.0)	24.0(53.3)	0.278

AML, acute myeloid leukemia; ALL, acute lymphoblastic leukemia; MDS, myelodysplastic syndrome; AA, aplastic anemia; ATG, antithymocyte globulin; GVHD, graft-versus-host disease; MNC, mononuclear cell; IQR, interquartile range.

**Figure 6:**
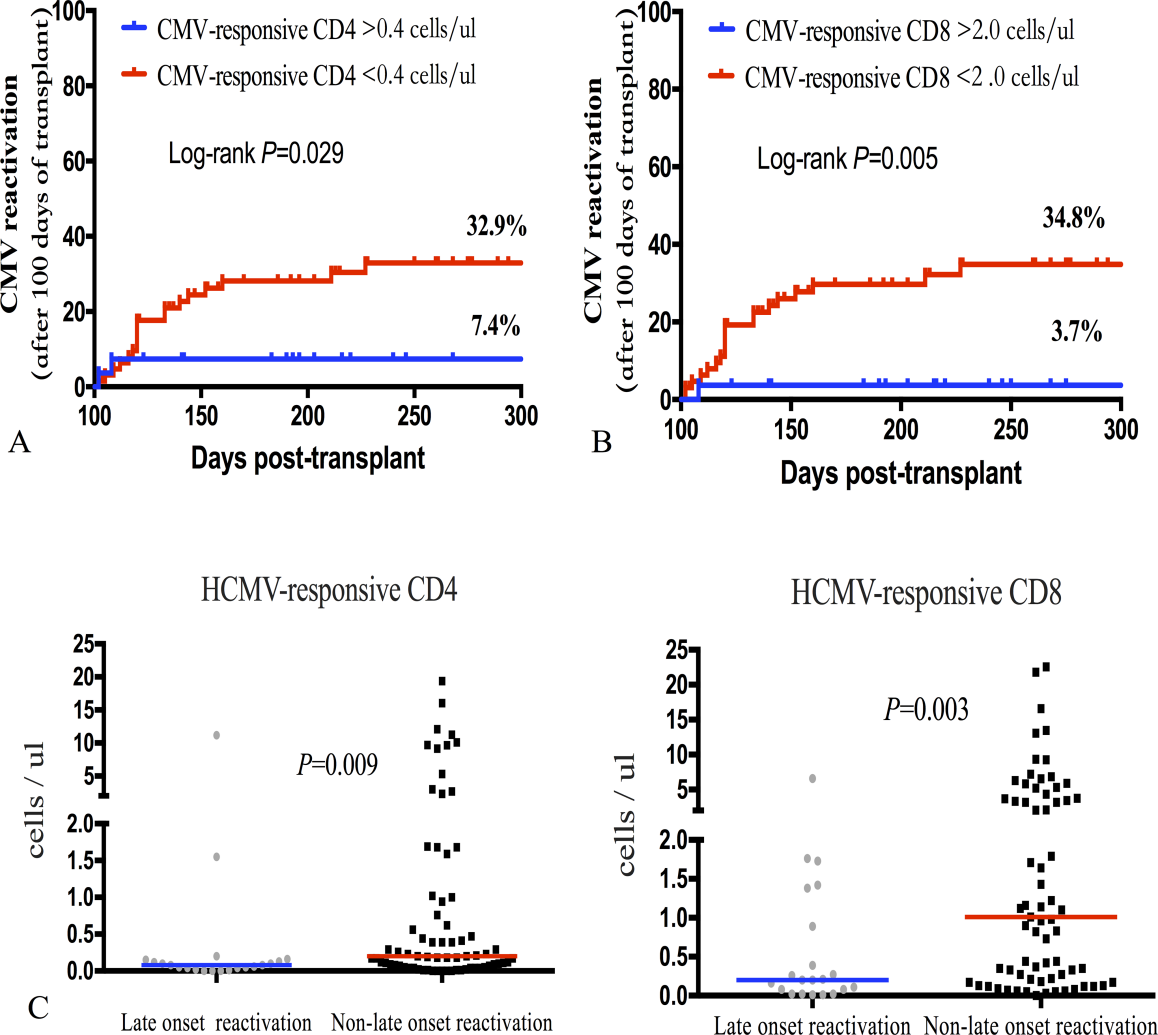
Incidence of late-onset (+100 days) CMV reactivation after allo-HSCT was significantly associated with CMV-responsive CD4+ and CD8+T cell levels 60 days after transplantation. **(A)** Incidence of late-onset CMV reactivation was calculated based on the recovery level of CMV-responsive CD4+T lymphocytes on day 60 after allo-HSCT; the incidence of late-onset CMV reactivation significantly increased in the group of CMV-responsive CD4+T cells (<0.40 cells/μL) (32.9% vs 7.4%; *P* = 0.029). **(B)** The incidence curve of late-onset CMV reactivation was calculated over time based on the recovery level of CMV-responsive CD8+T lymphocytes 60 days after allo-HSCT. The cumulative incidence of late CMV reactivation significantly increased in the CMV-responsive group (<2.00 CD8+T cells/μL) (34.8% vs 3.7%; *P* = 0.005). **(C)** The number of CMV-responsive CD4+T and CD8+T cells at day 60 after transplantation was calculated based on whether late-onset CMV reactivation occurred. The Mann–Whitney U test was used to assess differences in the distribution of CMV-responsive CD4+ and CD8+T cells in patients with and without late-onset CMV reactivation. The median level of each group is represented by the horizontal line in the scatter plot.

Patients who lacked CMV-responsive CD8+T cells day 60 post-transplantation had a significantly increased risk of CMV reactivation after day 100 of allo-HSCT (HR 9.932; *P* = 0.005). Similar to CMV-responsive CD8+T cells, the incidence of late-onset CMV reactivation significantly increased in patients with CMV-responsive CD4+T cells (<0.40/μL) 60 days after transplantation (**Figures 6A, B**). CMV-responsive CD4+T and CD8+T cells were significantly higher 60 days after transplantation in patients without late-onset CMV reactivation (*P* = 0.009, *P* = 0.003; **Figure 6C**). Therefore, the level of CMV-responsive T cells on day 60 after allo-HSCT can be used as a predictor of late-onset CMV reactivation after allo-HSCT to assist in judging the risk of late-onset CMV reactivation after transplantation and to help guide the monitoring frequency of CMV reactivation and make treatment decisions.

## 4 Discussion

Although some new antiviral drugs for the treatment of CMV infection have been developed and the clinical research and development of vaccines have made progress in recent years (16), CMV infection remains an important complication of transplantation and is associated with high mortality (13). Because the hematological and renal toxicity profiles of ganciclovir and foscarnet sodium, a preemptive treatment based on CMV–DNA monitoring, instead of prophylactic treatment, is usually adopted for allo-HSCT patients. In order to understand the reconstitution and function of CMV immunity after allo-HSCT and obtain reliable predictive factors for CMV infection, multi-parameter cytokine flow cytometry is generally employed in this study to study the immune response levels of CD4+ and CD8+T cells to CMV-PP65 overlapping peptides in patients after allo-HSCT, and the CMV-specific cellular immune function of the allo-HSCT donor was also included. The results showed that post-transplantation CMV reactivation is closely related to CMV-responsive CD4+T cells, and that CMV reactivation and RCMV are significantly reduced in patients with high levels of CMV-responsive CD4+T cells 30 days after transplantation. The level of CMV-responsive T cells 60 days after allo-HSCT has a high predictive value for late-onset CMV reactivation.

According to Pelak et al. (17), the reconstitution of CMV-specific CD8+T cells after allo-HSCT is significantly correlated with the anti-CMV infection therapy time. Gabanti et al. (12) reported that the reconstitution of CMV-specific CD8+T cells, which itself depends on the reconstitution of CMV-specific CD4+T cells, helps in controlling CMV infection. Therefore, both CD4+T and CD8+T cells are crucial for controlling CMV infection. CMV-specific cellular immune responses can be studied by stimulating T cells using overlapping peptides. The length, overlap, and reference sequence of the peptides influence the intensity and range of the T cell response. Peptides with 10–11 amino acid overlaps, totaling 15–20 amino acids, exhibit similar abilities to stimulate CD8+T cell responses. Shorter peptides are better for CD8+T cell responses, while the longer ones are more suitable for CD4+T cells (18). Our study demonstrated that the CMV-pp65 antigen-overlapping peptide pool displays good immunogenicity against both CD4+ and CD8+T cells.

The CMV serostatus of both the donor and recipient of allo-HSCT is associated with the reconstitution of CMV-specific cells. The CMV-specific CD8+T cells of the CMV-serotype-positive recipient are re-established faster than that for CD4+T cells (10, 19). Hence, it can be suggested that allo-HSCT conditioning cannot completely eliminate latent CMV in recipients, and that the lack of donor-derived CMV-specific lymphocytes delays CMV reconstitution and increases the risk of CMV reactivation. This study focused on the immune reconstitution of CMV-responsive T cells in patients with positive CMV serology. The level of CMV-responsive T cells in the peripheral blood of the donor does not exhibit any significant relationship with early specific cellular immunity reconstitution after allo-HSCT. Moreover, the reconstitution level of CMV-responsive T cells is related to the risk of CMV reactivation and late-onset CMV reactivation.

CMV latency in the CD34+ human hematopoietic stem cells, progenitor cells, and CD14+ monocytes can be reactivated when these cells differentiate to macrophages or dendritic cells in the bone marrow niche (20). The relative roles of the virus and host factors in initiating and maintaining CMV reactivation are still unclear. Changes in the host immune status may influence the expression of the viral genome by affecting signal transduction pathways, thereby facilitating the transition of the virus from the latent state to the lytic state (21). In the present study, CMV reactivation occurred 40.0 (32.3–50.0) days after allo-HSCT. This reactivation greatly reduced when the number of CMV-responsive T lymphocytes 60 days exhibited a large increase after allo-HSCT. Slobedman et al. (20) confirmed that the proportion of HCMV latently infected cells in the peripheral blood of healthy CMV serologically positive donors mobilized with G-CSF is extremely low (0.004–0.01% of PBMC). In addition, only approximately 2% of these latently infected cells could detect viral transcripts. No significant correlation was noted between the CMV-responsive CD4+T and CD8+T cells of donors and recipients. Hence, it can be suggested that in the early immune recovery balance process after allo-HSCT, regardless of the level of CMV-specific immunity of the donor, the CMV-specific T cell immunity of the recipient plays a key role in the self-reconstitution process, which needs to be further studied by increasing the sample size.

Immune cell subsets show significant variations in post-allo-HSCT reconstitution (22, 23). The absolute number of lymphocytes does not reflect the CMV-specific immune level (24). In addition, patients with CMV reactivation often exhibit an increase in the number of dysfunctional CMV-specific CD8+T cells. Therefore, functional CMV-specific T cells could more accurately reflect their control of CMV. We showed that the post-allo-HSCT reconstitution of CMV-responsive CD4+T cells was slower than that of CD8+T cells. However, the number of CMV-responsive CD8+T cells significantly increased 60 days after allo-HSCT. At the same time, a significant reduction in CMV reactivation was also noted, which demonstrates the key role of CMV-responsive T cells in controlling CMV reactivation. However, some scholars hold the opposite view, that the low levels of CMV reactivation can drive the selective reconstitution of CMV-specific immunity, and that the selective reconstitution of CMV-specific CD8+T cells prior to total T cell reconstitution is related to CMV reactivation (25). CMV-specific CD4+T and CD8+T cells amplify in response to CMV replication (26). Lugthart et al. (27) demonstrated that CMV reactivation has a long-term effect on the reconstitution of total T cells after transplantation, greatly increasing the number of CD8+ effector memory T cells. CMV reactivation is a risk factor for poor secondary graft function after allo-HSCT (28). Therefore, host immune surveillance function and immune response intensity play a major role in counterbalancing CMV reactivation.

The first 100 days after allo-HSCT are characterized by cellular immunodeficiency, with reduced numbers of cytotoxic lymphocytes, natural killer (NK) cells, and T cells of the adaptive immune system. Hence, patients are vulnerable to viral and fungal infections, such as CMV (4, 23). However, some patients can develop CMV infection even after 100 days of allo-HSCT. We investigated the early immune response of CD4+/CD8+T cells after allo-HSCT under stimulation by CMV-PP65 *in vitro*, and found that the reconstitution of CMV-responsive CD4+/CD8+T cells 60 days after allo-HSCT is closely related to the late-onset CMV reactivation. Early identification of high-risk patients with late-onset CMV reactivation is helpful in improving the risk stratification of late-onset CMV reactivation after allo-HSCT and improving outpatient management.

IFN-γ is mainly induced by activated T helper cell 1 (Th1) and cytotoxic T cells under antigenic stimulation. It plays a key role in both antiviral and anti-tumor immunity systems. However, IFN-γ produced by T cells cannot represent all CMV-specific immune responses (10). In this study, the lack of differentiation between clinical groups when IFN-γ-producing CMV-specific T cells were used as markers for predicting CMV reactivation also suggests that the T cell immune response at the level of one cytokine alone is too simplistic to assess the risk of CMV infection. Therefore, we simultaneously examined multiple functions of CMV-specific T cells. Patients lacking CMV-responsive CD4+T cells 30 days after allo-HSCT were at an increased risk of CMV reactivation (HR, 2.139; *P* < 0.001). Therefore, it is more beneficial to employ active antiviral treatment measures for such patients. A low level of CMV-responsive CD8+T cells (<2 cells/μL) 60 days after transplantation is a risk factor for late-onset CMV reactivation. Hence, the levels of these cells can be used as a predictor of late-onset CMV reactivation and for guiding the monitoring frequency and treatment strategies of late-onset CMV reactivation.

Because of the strict speciation specificity of CMV, it is difficult to use wild-type animal models to directly study the pathogenic mechanism and prevention of HCMV. Although the indicators identified in this study have application value in CMV reactivation, they need to be further verified to maintain the consistency of technical means and individual differences among patients. In addition, CMV reactivation is closely related to the incidence of GVHD, tumor recurrence, and non-relapse mortality, which warrants further exploration of the interaction between CMV reactivation and the immune system, as well as the mechanism and effect of CMV reactivation on the prognosis of patients with allo-HSCT.

## Data Availability

The original contributions presented in the study are included in the article/**Supplementary Material**. Further inquiries can be directed to the corresponding author.
