# Supplementary material for: Immune reconstitution of human cytomegalovirus-specific T lymphocytes after allogeneic hematopoietic stem cell transplantation and their predictive role in reactivation
Source: Front Immunol. 2025 Mar 12;16:1464096. doi: 10.3389/fimmu.2025.1464096 (PMC11937026; doi:10.3389/fimmu.2025.1464096)
Supplement: Supplementary file 1 [file Table1.docx]

Supplementary Material

**Table S1. Univariate analysis of factors associated with CMV reactivation after allo-HSCT**

| Characteristic | CMV reactivation | Non-CMV reactivation | *P*-value |
| --- | --- | --- | --- |
|  | (*n* = 88) | (*n* = 43) |  |
| Median age, (IQR) | 35.5（23.3-46.0） | 30.0（22.0-41.0） | 0.122 |
| Gender, male, *n* (%) | 40.0（45.5） | 18.0（41.9） | 0.421 |
| Disease type*, n* (%) |  |  | 0.207 |
| AML | 37.0（42.0） | 17.0（39.5） |  |
| ALL | 22.0（25.0） | 17.0（39.5） |  |
| MDS | 24.0（27.3） | 6.0（7.0） |  |
| AA | 5.0（5.7） | 3.0（7.0） |  |
| Transplantation type, *n* (%) |  |  | <0.001 |
| Unrelated donor | 7.0（8.0） | 3.0（7.0） |  |
| Matched related donor | 10.0（11.4） | 25.0（58.1） |  |
| Haploidentical donor | 71.0（80.7） | 15.0（34.9） |  |
| Prior ATG use, *n* (%) | 83.0（94.3） | 24.0（55.8） | <0.001 |
| Prior high-dose glucocorticoid, *n* (%) | 33.0（37.5） | 2.0（4.7） | <0.001 |
| III–IV aGVHD, *n* (%) | 19.0（21.6） | 2.0（4.7） | 0.012 |
| Leukemia relapse after allo-HSCT, *n* (%) | 15.0（17.0） | 6.0（14.0） | 0.801 |
| MNC dose, 10^8^/kg, median (IQR) | 11.2（8.5-15.1） | 10.4（9.0-12.3） | 0.525 |
| CD34+ cell dose, 10^6^/kg, median (*IQR*) | 2.7（2.4-3.2） | 2.4（2.2-2.6） | 0.001 |
| Donor and recipient in the same blood type | 56.0（63.6） | 23.0（53.5） | 0.337 |
| CMV-CD4+T cells at 30 days, /μL | 0.091(0.008-0.483) | 0.207(0.095-1.029) | 0.005 |
| CMV-CD8+T cells at 30 days, /μL | 0.258(0.042-1.207) | 0.486(0.172-1.339) | 0.07 |

**Table S2. Risk factors for CMV reactivation in patients after allo-HSCT based on multivariate analysis**

| Characteristic | OR | | 95% CI | *P*-value |
| --- | --- | --- | --- | --- |
| Transplantation type | | 0.505 | 0.245-1.039 | 0.064 |
| Prior ATG use | | 7.368 | 1.987-27.316 | 0.003 |
| Prior high-dose glucocorticoid | | 5.717 | 1.074-30.438 | 0.041 |
| CD34+ cell dose > 2.59 × 10^6^/kg, | | 1.765 | 0.693-4.495 | 0.234 |
| CMV–CD4 > 0.14/μL | | 0.640 | 0.250-1.637 | 0.352 |
| III–IV aGVHD | | 3.435 | 0.408-28.931 | 0.256 |

OR, odds ratio; CI, confidence interval.

**Table S3. Risk factors for the reconstitution of CMV-responsive CD8+T cells 60 days after allo-HSCT based on multivariate analysis**

| Characteristic | OR | 95%CI | P-value |
| --- | --- | --- | --- |
| Age | 0.957 | 0.920-0.995 | 0.029 |
| MNC dose | 0.954 | 0.864-1.055 | 0.361 |

**Table S4. Factors influencing the reconstitution of CMV-responsive CD4+T cells 60 days after allo-HSCT**

| Characteristic | High levels of CMV–CD4 | Low levels of CMV–CD4 | P-value |
| --- | --- | --- | --- |
|  | (n=27) | (n=65) |  |
| Median age, (IQR) | **37.0（34.0-50.0）** | **33.0（19.5-44.5）** | **0.019** |
| Gender, male | **11.0（40.7）** | **28.0（43.1）** | **0.512** |
| Disease type, *n* (%) |  |  | **0.347** |
| AML | **11(40.7)** | **24(36.9)** |  |
| ALL | **9(33.3)** | **19(29.2)** |  |
| MDS | **6(22.2)** | **18(27.7)** |  |
| AA | **1(3.7)** | **4(6.2)** |  |
| Transplantation type, *n* (%) |  |  | **0.072** |
| Unrelated donor | **2(7.4)** | **6(9.2)** |  |
| Matched related donor | **10(37.0)** | **10(15.4)** |  |
| Haploidentical donor | **15(55.6)** | **49(75.4)** |  |
| Prior ATG use, *n* (%) | **22(81.5)** | **58(89.2)** | **0.247** |
| Prior high-dose glucocorticoid, *n* (%) | **7.0（25.9）** | **25.0（38.5）** | **0.182** |
| Grade III–IV aGVHD, *n* (%) | **5.0(18.5)** | **18.0(27.7)** | **0.258** |
| Donor and recipient of the same blood type, *n* (%) | **22(81.5)** | **37(56.9)** | **0.018** |
| MNC dose, 10^8^/kg, median (IQR) | **11.75（9.27-14.31）** | **10.00（8.05-14.10）** | **0.289** |
| CD34+ cell dose, 10^6^/kg | **2.61（2.33-3.73）** | **2.62（2.32－3.03）** | **0.398** |
| Days to neutrophil engraftment, median (IQR) | **12.0（11.0-14.0）** | **12.0（11.0-15.0）** | **0.338** |
| Days to platelet engraftment, median (IQR) | **14.0（12.75-16.0）** | **17.0（14.75-20.0)** | **0.027** |
| Late-onset CMV reactivation, n (%) | **2.0(7.4)** | **19.0(29.2)** | **0.018** |

**Table S5. Risk factors for the reconstitution of CMV-responsive CD4+T cells 60 days after allo-HSCT based on multivariate analysis**

| Characteristic | OR | 95%CI | P-value |
| --- | --- | --- | --- |
| Age | 0.967 | 0.912-1.026 | 0.270 |
| Donor and recipient of the same blood type | 19.456 | 1.767-214.214 | 0.015 |
| Days to platelet engraftment | 1.220 | 0.970-1.534 | 0.090 |
